# Feasibility of Pressurized Intraperitoneal Aerosol Chemotherapy (PIPAC) in a Rabbit Model of Peritoneal Metastases: PIPALIM Project

**DOI:** 10.1245/s10434-025-17251-7

**Published:** 2025-04-25

**Authors:** Sylvia M. Bardet, Marie-Laure Perrin, Valentin David, Catherine Yardin, Alain Chaunavel, Karine Durand, Gaelle Maillan, Aymeric Rouchaud, Sylvaine Durand Fontanier, Abdelkader Taibi

**Affiliations:** 1 XLIM, UMR, CNRS 7252, University Limoges, Limoges, France; 2 Digestive Surgery Department, Dupuytren University Hospital, Limoges, France; 3 Cytology and Histology Department, Dupuytren University Hospital, Limoges, France; 4 Translational Research and Innovation Platform in Oncology, Pathology Department, Dupuytren University Hospital, Limoges, France; 5 Pharmacy Department, Dupuytren University Hospital, Limoges, France; 6 Radiology Department, Dupuytren University Hospital, Limoges, France; 7 EMIS Research, Dupuytren University Hospital , Limoges, France

**Keywords:** Peritoneal metastases, PIPAC, Rabbit model, Intraperitoneal chemotherapy, Cancer

## Abstract

**Background:**

Animal models are essential for testing new pressurized intraperitoneal aerosol chemotherapy (PIPAC) protocols; however, no immunocompetent animal model of peritoneal surface malignancies (PSMs) treated with PIPAC has been established. This study evaluated the feasibility, safety, and oncological efficacy of PIPAC in a rabbit PSM model.

**Methods:**

The study was conducted in two phases: (1) Feasibility Assessment: Three healthy rabbits underwent three consecutive PIPAC procedures (saline) at weekly intervals. The rabbits’ well-being, morbidity, mortality, and histological changes were monitored. (2) Treatment Phase: Rabbits with PSM were treated with PIPAC using oxaliplatin, cisplatin-doxorubicin, or saline. Parameters such as animal well-being, ascites volume, morbidity, Peritoneal Cancer Index (PCI), histological response (Peritoneal Regression Grading Score [PRGS]), tumor cell proliferation/apoptosis, and circulating tumor DNA (ctDNA) levels were assessed.

**Results:**

PIPAC was feasible and safe, with no increased morbidity or mortality. PIPAC demonstrated antitumor efficacy with lower PCI (control 21.6 vs. oxaliplatin 9.2 vs. cisplatin-doxorubicin 10.2; *p* < 0.001), improved histological response (PRGS: control 3.38 vs. oxaliplatin 1.95 vs. cisplatin-doxorubicin 1.85; *p* = 0.01), and reduced tumor cell proliferation (control 5.3% vs. oxaliplatin 0.82% vs. cisplatin-doxorubicin 0.62%; *p* < 0.0001). ctDNA levels showed promise for monitoring treatment response, warranting further investigation.

**Conclusion:**

This study confirms the feasibility and effectiveness of PIPAC with oxaliplatin or cisplatin-doxorubicin in rabbits with PSM. The model provides a foundation for future research on PIPAC protocols and related treatments.

**Supplementary material:**

The online version of this article (10.1245/s10434-025-17251-7) contains supplementary material, which is available to authorized users.

Pressurized intraperitoneal aerosol chemotherapy (PIPAC) is prescribed exclusively for patients with unresectable peritoneal surface malignancies (PSMs).^1^ This technique reduces the chemotherapy dose administered intraperitoneally by nebulizing it into an aerosol form, ensuring even distribution within the various peritoneal recesses and across any adhesions. The abdominal pressure created during laparoscopy enhances the penetration of chemotherapy into the peritoneum. Additionally, PIPAC can be combined with electrostatic therapy, which accelerates the stabilization of intraperitoneal chemotherapy and further improves its penetration into the PSMs while reducing the duration of the procedure.^2^ The main chemotherapy protocols commonly used in clinical practice for patients with PSMs include platinum-based agents such as oxaliplatin and cisplatin combined with doxorubicin.^3,4^

However, several questions remain unanswered regarding the choice of spray nozzles, various chemotherapy protocols, and new targeted therapies.^5–8^ These uncertainties call for further investigation, and experimental research could help address these issues. The importance of animal models in cancer research, particularly for PSMs, is well established. Animal models have played an indispensable role in studying the pathophysiology of PSMs and have significantly contributed to advances in clinical practice;^9^ however, due to their small size and limited abdominal cavity, small rodents such as mice are not suitable for performing PIPAC.

The rabbit model for PSMs offers an interesting alternative. Classified as a ‘large animal’ model, the rabbit is comparable in size to a newborn infant and has a peritoneal cavity large enough to accommodate laparoscopy with high pneumoperitoneum.^10,11^ Rabbits are relatively easy to breed, have a longer lifespan than rodents, and their peritoneal cavity size allows for complex surgical procedures such as PIPAC to be performed. To date, no PIPAC procedures have been carried out on a rabbit model of peritoneal cancer.

The first objective was to evaluate the feasibility and safety of the PIPAC procedure by using a model with a larger intraperitoneal cavity compared with the previously used rat models. The second objective was to assess the therapeutic response of PSMs following treatment with pressurized intraperitoneal chemotherapy (oxaliplatin and cisplatin-doxorubicin) in an immunocompetent rabbit model of PSMs.

## Methods

### Animal selection

Female New Zealand rabbits, aged 4 months (weighing approximately 2 kg) were purchased from an approved supplier (Gontran Achard Breeding Center for Sale with Controlled Health Status [CEGAV SSC], Argenvilliers, France). This study was carried out with the ethical approval from the French National Ministry of Research (Authorization of Projects for Scientific Purposes #28502-2020120314287244) and complied with ethical regulations (decree no. 2001-131, European directive 86-609-CEE 1986) and the ARRIVE guidelines 2.0.^12^

### Preliminary Study: Safety of Physiological Serum Pressurized Intraperitoneal Aerosol Chemotherapy (PIPAC)

#### Experimental Protocol for PIPAC in Rabbits

**Fig. 1:**
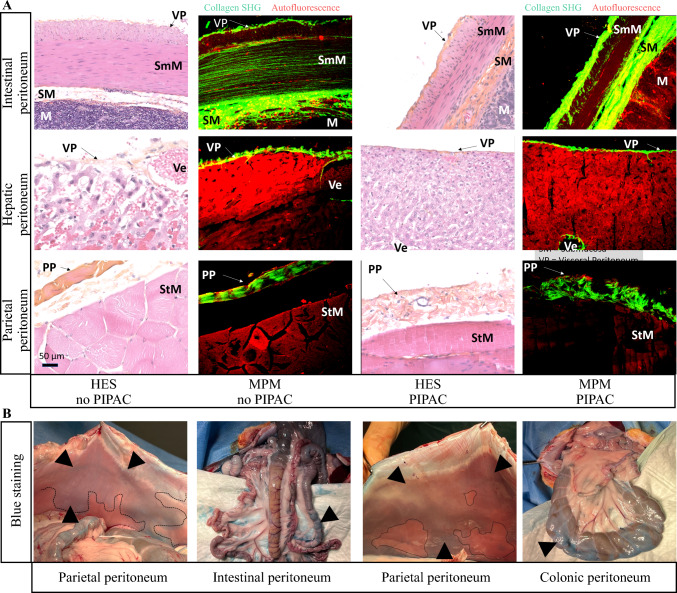
**A** HES staining and multiphoton imaging of rabbit visceral and parietal peritoneum with and without PIPAC. Scale bar in (a) = 50 µm. **B** Photographs of blue staining after PIPAC in intra-abdominal cavities illustrate the extent of nebulization diffusion (black arrowheads). *M* muscularis, *PP* parietal peritoneum, *SHG* second harmonic generation, *StM* striated muscle, *SM* submucosa, *VP* visceral peritoneum, *SmM* smooth muscle, *Ve* blood vessel, *HES* hematoxylin-eosin-saffron, *PIPAC* pressurized intraperitoneal aerosol chemotherapy

After a 1-week acclimatization period, three rabbits were treated by physiological serum PIPAC, while a fourth rabbit served as a control to obtain organ biopsies without PIPAC treatment. The rabbits were fed a standard diet and housed under a 12-h light/dark cycle. The animals were euthanized at the end of the experiment and immediately autopsied. The PIPAC experimental procedure in rabbits was based on clinical treatment protocols.^13^ The animals were anesthetized with an intramuscular injection of ketamine (Renaudin Laboratory, Itxassou, France) at a dose of 15–20 mg/kg combined with xylazine (Rompun 2%; Elanco, Cuxhaven, Germany) at 4 mg/kg and acepromazine (Calmivet; Vetoquinol, Tarare, France) at 0.85 mg/kg. The abdomen was shaved and disinfected. An open laparoscopy was performed under visual control, and a 12 mm balloon trocar was inserted. Abdominal exploration was carried out using an optical camera (zero-degree angle) after obtaining a constant capnoperitoneal pressure of 8 mmHg. A second 10 mm trocar was placed in the midline and secured. The nebulizer nozzle (770–12, Reger Medizintechnik, Rottweil, Germany) was then connected to the high-pressure injector via a high-pressure line and inserted into the abdominal cavity through the trocar. The abdominal cavity was checked for tightness (absence of CO₂ leakage). The high-pressure injector was then activated with the injection parameters set at a flow rate of 0.8 mL/s and a maximum upstream injection pressure of 20 bars (electronic supplementary material [ESM] Figs. 1a–c).

The PIPAC procedure (50 mL of physiological serum – 0.9% NaCl) was performed under clinical practice conditions over a period of 30 min on days 8, 15, and 21.^13^ On day 26, indigo carmine (10 mg, Carmyne^®^; SERB, France) and 0.9% NaCl (50 mL) were mixed and injected via the PIPAC procedure into the peritoneal cavity for 30 min. An abdominal cone beam computed tomography (CT; Artis Icono, Siemens Healthineers, France) was performed after the third PIPAC. Euthanasia was then carried out using an intravenous injection of 1–2 mL of T-61 (embutramide 200 mg/mL + mebezonium 26.92 mg/mL + tetracaine 4.39 mg/mL). A xiphopubic laparotomy was performed and two researchers visually assessed the distribution and intensity of blue staining on the visceral and parietal peritoneum of the rabbits (ESM Fig. 1d).

#### Criteria Analysis

The well-being score was analyzed, including assessments of physical activity, facial expressions, resting posture, appearance, nutrition, hydration, and responsiveness to stimuli (scored from 0 to 10). Morbidity and mortality were also evaluated. Histological analysis (hematoxylin-eosin-saffron [HES] staining, Tissue-Tek Prisma™, Sakura Finetek, The Netherlands) and microscopic multiphoton analysis of the liver and visceral and parietal peritoneum were conducted by two expert surgeon researchers in the field.^14^

### Oxaliplatin and Cisplatin-Doxorubicin PIPAC Study

This experimental feasibility study in a rabbit model with peritoneal metastases compared the effects of PIPAC oxaliplatin (*n* = 5) with PIPAC cisplatin-doxorubicin (*n* = 5) and a control group treated with physiological saline (*n* = 5).

#### Rabbit Model of Peritoneal Metastasis

The VX2 tumor was serially transmitted into the hind leg of New Zealand white rabbits (*n*= 2) following the procedure described by Mei et al.^10^ and Pascale et al.^15^ After 15 days, the tumors were placed into cell suspension and implanted via mini laparotomy in the stomach, omentum, visceral peritoneum (small intestine), and parietal peritoneum using an 18G spinal needle (Becton Dickinson, Franklin Lakes, NJ, USA). The tumors were allowed to grow for 8 days before treatment.

#### Experimental Design

The body surface area of a 3.5 kg rabbit is 0.233 m^2^. Three groups of five rabbits were randomized on day 8 after tumor implantation into the following groups: control (PIPAC with 50 mL physiological saline); oxaliplatin (92 mg/m^2^); and cisplatin (10.5 mg/m^2^) – doxorubicin (2.1 mg/m^2^).

The PIPAC procedures were performed on days 8, 15, and 21 after tumor implantation, and euthanasia was performed on day 26 (ESM Fig. 1e). An abdominal CT scan was performed prior to each PIPAC procedure and euthanasia.

#### Criteria Analysis

The well-being score was calculated before each PIPAC procedure. At each PIPAC, the volume of ascites was measured and the Peritoneal Cancer Index (PCI) was visually calculated. Biopsies of at least three nodules were taken and fixed in 4% paraformaldehyde and embedded in paraffin. Sections (4-μm-thick) were cut and stained with HES using an automated Benchmark XT instrument (Roche), for mouse monoclonal Ki67/MKI67 antibody (8D5, NBP2-22112, Bio-Techne, France), and TUNEL assay kit (HRP DAB, ab206386, Abcam, France). The histological response (HR) score is visually assessed according to the Peritoneal Regression Grading Score (PRGS). A surface index based on Ki-67 and TUNEL status was established to compare proliferation and dead cell rates (%) with Fiji/ImageJ (National Institutes of Health). Circulating DNA isolation and quantification were performed as previously described.^16^ For DNA isolation, we followed the manufacturer’s instructions regarding the use of RNA carrier but omitted the concentration step using Agencourt AMPure beads (Beckman Coulter). Quantification of VX2 ctDNA was performed as previously described, with the following modifications: quantitative polymerase chain reaction (qPCR) was carried out in triplicate in 20 µL reactions, each consisting of MeltDoctor HRM Master Mix (Applied Biosystems), 200 nM oligos, and 1.5 µL of purified DNA.

#### Statistical Analysis

Data were analyzed using a one- or two-way repeated measure of analysis of variance (ANOVA), with post hoc tests to locate the source of significant differences using Bonferroni or Sidak corrections for multiple comparisons (GraphPad Prism). The results are presented as means with standard deviation (SD). Asterisks in the figures indicate statistically significant differences compared with controls where the probability of falsely rejecting the null hypothesis was <5% (*p* < 0.05).

## Results

### Preliminary Study: Safety of Physiological Serum PIPAC

The well-being scores of the three rabbits following the PIPAC procedures were <2 points due to weight loss. A small intestine perforation occurred during PIPAC 3 in one rabbit, but it was successfully sutured without postoperative complications. No rabbits died during the preliminary experiment.

Histological analysis revealed that the peritoneum following PIPAC contained as much collagen as that of control rabbits. In the same way, no additional tissue inflammation was observed in the peritoneum or liver parenchyma (Fig. 1a).

Diffusion of the blue dye administered by PIPAC was homogeneous across the parietal peritoneum, as well as the peritoneum of the small intestine and colon (Fig. 1b); however, diffusion was weaker in the diaphragmatic dome and the pouch of Douglas.

### Therapeutic PIPAC Study: Oxaliplatin and Cisplatin-Doxorubicin Treatments

**Fig. 2:**
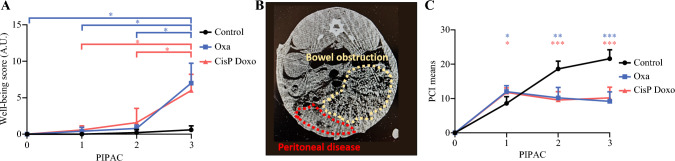
**A** Impact of PIPAC-Oxa and PIPAC-CisP Doxo on rabbit well-being score versus control group. **B** Image of an abdominal CT scan at day 26 in a control rabbit revealed advanced peritoneal disease and bowel obstruction. **C** Mean score of the PCI in each treatment group. *PIPAC* pressurized intraperitoneal aerosol chemotherapy, *CT* computed tomography, *PCI* Peritoneal Cancer Index, *Oxa* oxaliplatin, *CisP Doxo* cisplatin-doxorubicin (mean ± standard deviation), * *p* < 0.05, ** *p *< 0.01, *** *p* < 0.001

*The well-being score* increased in all groups (<2 points) but these scores were higher in the groups treated with chemotherapy after PIPAC 3 compared with the control group (mean ± SD 7 ± 2.7 and 6 ± 2.3 vs. 0.6 ± 0.5, F(6,36) = 9.40, *p* < 0.001) [Fig. 2a]. Weight loss was greater in the treated rabbits with either oxaliplatin (16%, *p* = 0.018) or cisplatin-doxorubicin (16%, *p* < 0.001) than in the control group (7.5%, *p* = 0.051).

*Radiological response*: The abdominal CT scan was difficult to interpret on day 8 and the radiological response could not be analyzed; however, after the three PIPACs, the peritoneal metastasis were visible in the control group (Fig. 2b).

*PCI score between PIPAC 1 and PIPAC 3:* Tumoral progression was heterogeneous across the three groups. The PCI score decreased slightly in the oxaliplatin group, as well as in the cisplatin-doxorubicin group, between PIPAC 1 and PIPAC 3 (oxaliplatin: 12 ± 1.7 vs. 9.2 ± 2.8, *p *= non-significant; cisplatin-doxorubicin: 11.8 ± 1.3 vs. 10.2 ± 3.1, *p =* non-significant) and was significantly higher in the control group (8.6 ± 1.9 vs. 21.6 ± 2.6, *p* = 0.007). The PCI score after PIPAC 3 was significantly higher in the control group compared with the oxaliplatin and cisplatin-doxorubicin groups (21.6 ± 2.6 vs. 9.2 ± 2.8 vs. 10.2 ± 3.1, *p* < 0.001) [Fig. 2c]. The volume of ascites was <50 mL in all animals, with no statistically significant differences observed.

**Fig. 3:**
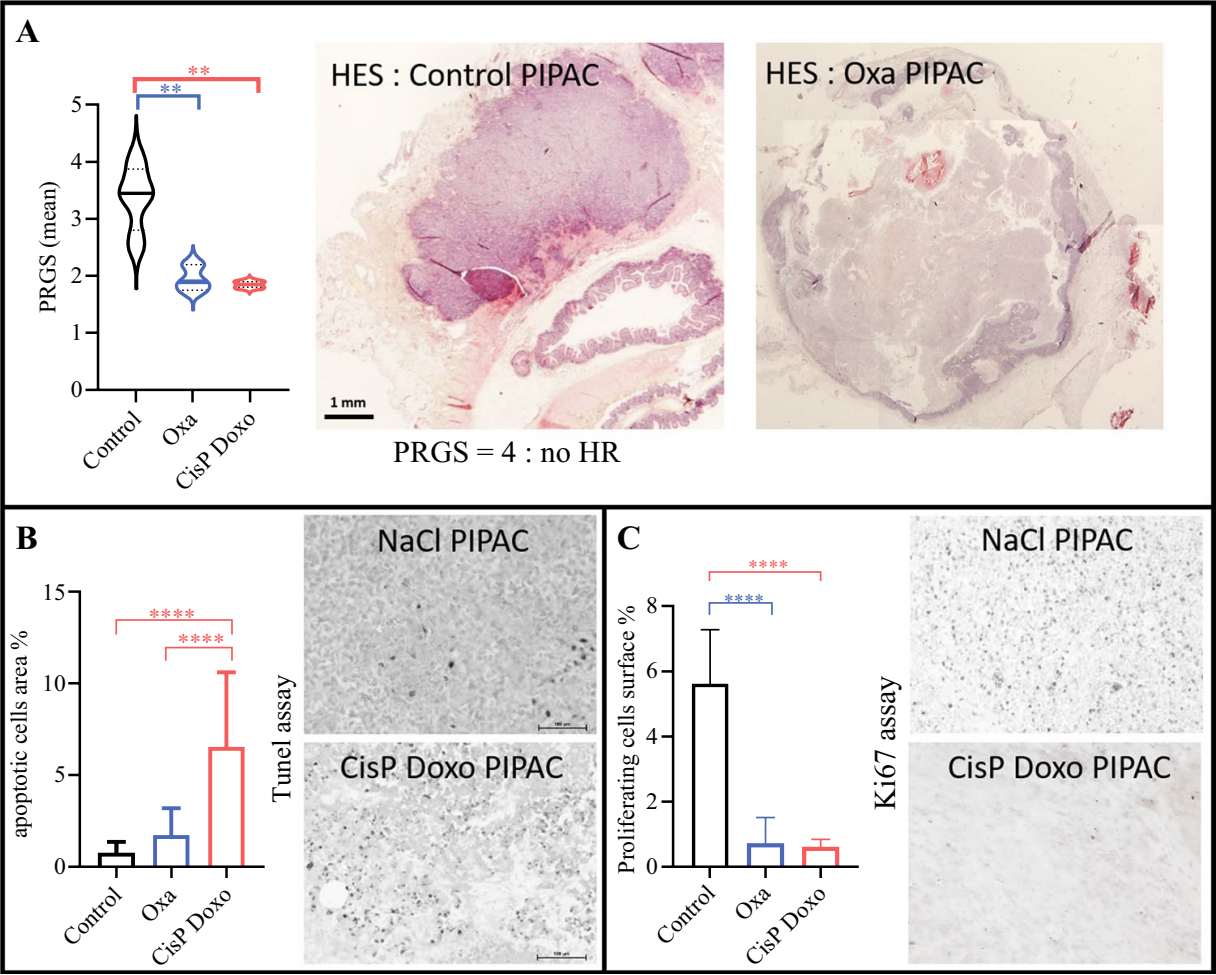
Histological analyses at D26 after the third PIPAC treatment. **A** HR was evaluated based on the PRGS. **B** Cell death by apoptosis was assessed using the TUNEL assay. **C** Cell proliferation was evaluated via anti-Ki67 immunostaining. Scale bars are in included in each image. *PIPAC* pressurized intraperitoneal aerosol chemotherapy, *HR* histological response, *PRGS* Peritoneal Regression Grading Score, *HES* hematoxylin-eosin-saffron staining, *Oxa* oxaliplatin, *CisP Doxo* cisplatin-doxorubicin (mean ± standard deviation), * *p* < 0.05, ** *p *< 0.01, *** *p* < 0.001, **** *p* < 0.0001

*Histological response, cell proliferation and death:* We observed a statistically significant difference in PRGS means between the treated groups (oxaliplatin: 1.95 ± 0.23; cisplatin-doxorubicin: 1.85 ± 0.07) and the control group (3.38 ± 0.58, *p* = 0.01) [Fig. 3a]. The oxaliplatin and cisplatin-doxorubicin treatments demonstrated comparable HR outcomes. PIPAC with cisplatin-doxorubicin induced a pronounced apoptotic wave at D26, as demonstrated by the TUNEL assay, compared with NaCl and oxaliplatin treatments (control: 0.78 ± 0.58; oxaliplatin: 1.76 ± 1.45; cisplatin-doxorubicin: 6.55 ± 4.07, *p* < 0.0001) [Fig. 3b]. The proliferative index (Ki67+ cells) was significantly higher (*p* < 0.0001) in the control group (NaCl: 5.3% ± 0.4) compared with the chemotherapy-treated groups (oxaliplatin 0.82% ± 0.22; cisplatin-doxorubicin: 0.62% ± 0.13) (Fig. 3c).

**Fig. 4:**
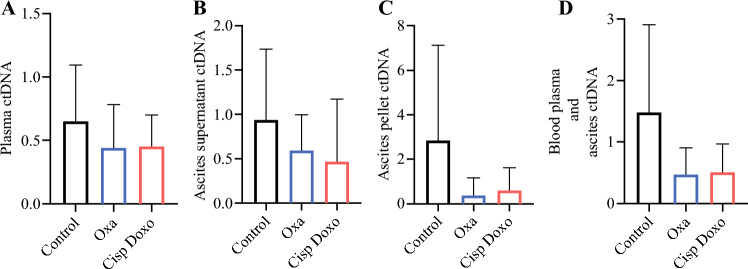
ctDNA levels (mean ± standard deviation) in **A** plasma, **B** abdominal ascites supernatant, and **C** ascites cell pellet are higher but not statistically significant in the untreated rabbit group (control PIPAC) compared with the treated groups (PIPAC with oxaliplatin or cisplatin-doxorubicin). **D** The mean of the three previous parameters shows a similar trend, although the differences are still not statistically significant. *CtDNA* circulating tumor DNA, *Oxa* oxaliplatin, *CisP Doxo* cisplatin-doxorubicin, *PIPAC* pressurized intraperitoneal aerosol chemotherapy

ctDNA levels were measured in plasma (Fig. 4a), abdominal ascites supernatant (Fig. 4b), and ascites cell pellet (Fig. 4c), and means were higher but not statistically significant in the untreated rabbit group (control PIPAC) compared with the treated groups (PIPAC with oxaliplatin or cisplatin-doxorubicin). The means of the three previous parameters showed a similar trend, although the differences were still not statistically significant (control: 1.48 ± 1.43; oxaliplatin: 0.48 ± 0.43; cisplatin-doxorubicin: 0.51 ± 0.46) (Fig. 4d).

## Discussion

This experimental study confirmed the feasibility and safety of PIPAC for the first time in a rabbit model with PSMs. It also highlighted the oncological potential of this innovative approach for intraperitoneal chemotherapy administration in this context. This immunocompetent model will establish a robust framework for future experiments on PIPAC, enabling the investigation of unresolved questions, such as comparisons of medical devices, chemotherapy protocols, and related factors.

Various models of PSMs have been described in the literature, the majority of which are mouse models involving intraperitoneal injection of tumor cells.^9,17,18^ Rat models, with the advantage of a larger intraperitoneal cavity size, have been successfully utilized for the PIPAC technique, which has been described and published with promising results.^19^ However, these models are immunocompromised, which makes oncological outcomes more challenging to translate, given the now well-established importance of cancer-microenvironment interactions in treatment response.^20–22^

In our study, PIPAC was performed under conditions closely resembling those in humans, with a slightly lower intra-abdominal pressure (8 mmHg vs. 12 mmHg in humans). This ensures the relevance of the technique, as intra-abdominal pressure is believed to play a significant role in enhancing chemotherapy penetration. Increased intraperitoneal pressure does not affect distribution patterns but leads to greater penetration depth of doxorubicin in a sheep model.^23^ Perioperative and postoperative morbidity were considered low, with one intestinal injury sutured without consequences for the rabbit. This aligns with conclusions from studies on PIPAC, where major perioperative and postoperative morbidity is estimated at <10%.^1^

Nevertheless, the animal welfare score was lower in the chemotherapy treated groups, primarily due to weight loss, although it remained below the threshold value established by the Ethics Committee. This weight loss is likely attributable to intraperitoneal chemotherapy rather than the disease itself, based on the results observed in the control group. Therefore, it will be necessary to limit the duration of the model to no more than 1 month of experimentation, and restrict the number of PIPAC procedures to a maximum of three. This is not a limitation as, in clinical practice, the evaluation of response to PIPAC procedures is typically conducted after the third PIPAC. During our procedures, no significant adhesions were detected that could have limited access to the abdominal cavity, one of the main reasons for discontinuing PIPAC in routine practice.^24,25^

The criteria for evaluating therapeutic response after PIPAC used in this study are the same as those applied in routine practice. All results are consistent with a better response in the two groups treated with PIPAC, regardless of the chemotherapy used (oxaliplatin or cisplatin-doxorubicin). The PCI was significantly lower in the groups treated with PIPAC, with an improved HR according to the PRGS. Proliferation and cell death were consistently decreased in treated rabbit compared with the non-treated group. Radiological response could not be assessed in this model after PIPAC treatment, likely due to the absence of contrast agent injection (known for its nephrotoxicity) and fecal stasis. However, in the control group, tumors were much more visible, exceeding 1 cm, and were located on the parietal peritoneum of the anterior wall. It will be necessary to develop a more suitable imaging protocol to evaluate this type of response in future experiments.

The measurement of circulating tumor DNA (ctDNA) is innovative and increasingly being evaluated in cancers, particularly for PSMs.^26^ In this feasibility study, ctDNA could be measured after treatment in all treated and untreated groups. This result is very interesting and will pave the way for future studies on the subject. Moreover, it seems that the ctDNA levels are lower after PIPAC, but the non-significant results due to the small number of rabbits encourage us to conduct further studies to confirm this finding.

The main limitation of this experimental study is the number of animals used per group, which can be easily justified in future studies presented to the Ethics Committee. This experimental study demonstrates that PIPAC is feasible and effective in this robust rabbit model with PSM and will open new avenues for exploring the translation of these results to humans more quickly.

## Conclusion

This experimental study confirms that PIPAC using oxaliplatin or cisplatin-doxorubicin is feasible and effective in a rabbit model with PSM. Thus, this model can now be used for future studies comparing PIPAC chemotherapy protocols or addressing other questions related to this new therapy.

## Supplementary material

Below is the link to the electronic supplementary material.

Supplementary file 1.
