# Supplementary material for: Feasibility of Pressurized Intraperitoneal Aerosol Chemotherapy (PIPAC) in a Rabbit Model of Peritoneal Metastases: PIPALIM Project
Source: Ann Surg Oncol. 2025 Apr 25;32(8):6050–7. doi: 10.1245/s10434-025-17251-7 (PMC12222299; doi:10.1245/s10434-025-17251-7)

**SUPPLEMENTARY FIGURE 1:** The main steps of the PIPAC procedure in rabbits are similar to those in clinical practice, including the use of instruments, trocar sizes, and safety protocols. A sterile environment is established after shaving and disinfecting the abdomen (A). Open laparoscopy is performed at two sites (B) to introduce the trocars for capnoperitoneal pressure (8 mmHg in rabbits versus 12 mmHg in humans), the nebulizer, and the camera (C).

Illustration of timeline and experimental procedures of PIPAC in the rabbit for (D) the preliminary study about safety of physiological serum PIPAC and (E) oxaliplatin and cisplatin-doxorubicin PIPAC study.


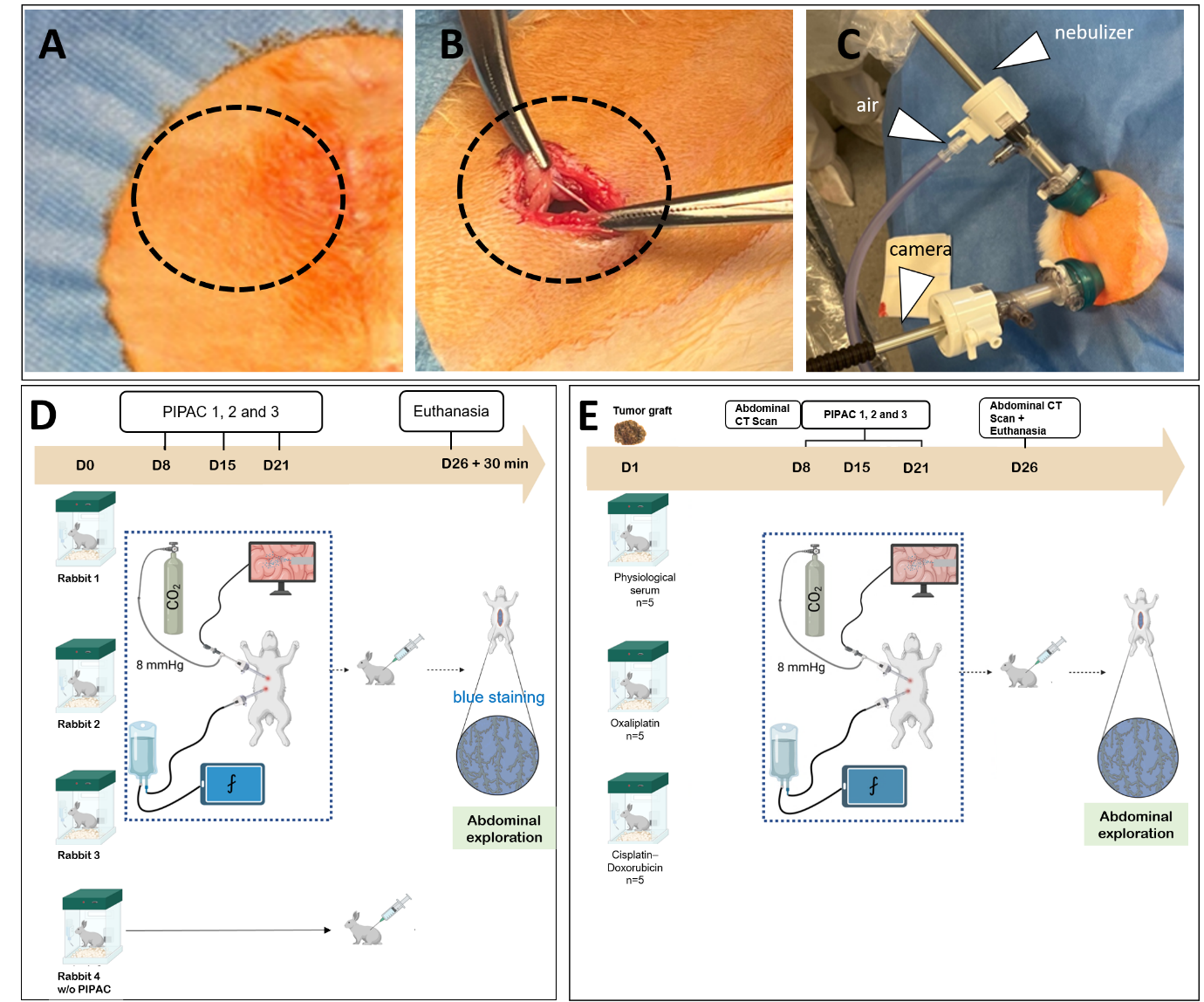

Supplement: Supplementary file 1 [file 10434_2025_17251_MOESM1_ESM.docx]
